# High-Dose Chemotherapy with Stem Cell Rescue in Desmoplastic Small Round Cell Tumor: A Single-Institution Experience and Review of the Literature

**DOI:** 10.1155/2018/1948093

**Published:** 2018-05-06

**Authors:** Kayleen Bailey, Michael Roth, Daniel Weiser, Jonathan Gill

**Affiliations:** ^1^Division of Pediatric Hematology Oncology, Memorial Sloan Kettering Cancer Center, New York Presbyterian Hospital Cornell, New York, NY, USA; ^2^Division of Pediatrics, University of Texas MD Anderson Cancer Center, Houston, TX, USA; ^3^Division of Pediatric Hematology, Oncology, Marrow and Blood Cell Transplantation, The Children's Hospital at Montefiore, Bronx, NY, USA

## Abstract

**Purpose:**

Desmoplastic small round cell tumor (DSRCT) is a rare cancer that predominantly affects males averaging 21 years of age at the time of diagnosis. We describe four cases from our institution and place them within the context of a comprehensive review of the literature.

**Patients and Methods:**

Study population included any patient who received treatment at Children's Hospital at Montefiore (CHAM) with histologic diagnosis of DSRCT. A search of the electronic databases PubMed, Cochrane Central Register of Controlled Trials, MEDLINE, and EMBASE for the terms “desmoplastic” AND “small” AND “round” AND “cell” AND “tumor” was performed.

**Results:**

One CHAM patient died of disease at 39 months, one patient has relapsed disease at 40 months, and two patients have no evidence of disease at 60 and 91 months. In the literature review, the 3-year OS was 36% and 5-year OS was 13%. There was a statistically significant difference in OS between no transplant and SCT in remission (*p*=0.004); however, there was no difference between no transplant and SCT not in remission (*p*=0.23).

**Conclusion:**

Given the poor prognosis in DSRCT, this study supports further prospective research into the possible benefit of consolidation of autologous SCT in patients with DSRCT who are in remission, with the alternative inference that these patients in remission may fare well without SCT. Our retrospective review of the literature does not support SCT for patients who are not in remission.

## 1. Introduction

Desmoplastic small round cell tumor (DSRCT) is a rare cancer which predominantly affects males averaging 21 years of age at the time of diagnosis [1]. Since its first description in 1991, approximately 500 cases have been published worldwide [2, 3]. Depending on the size of the tumor, patients can present with a range of symptoms: nausea, emesis, abdominal pain or distention, constipation, bowel obstruction, and acute renal failure. DSRCT has been described histologically as clusters of small round blue cells with polyphenotypic differentiation within a desmoplastic framework [4]. Because it contains epithelial, neural, and mesenchymal features, the tumor stains positive for desmin, keratin, vimentin, and epithelial membrane antigen. DSRCT is characterized by the fusion of the Wilms tumor (*WT1*) gene and the Ewing sarcoma (*EWS*) gene to form the t(11;22)(p13;q12) fusion, resulting in the upregulation of growth factors on the *EWS* gene and loss of tumor suppressor function of *WT1* [5]. The *EWS/WT1* fusion gene can be identified utilizing polymerase chain reaction (PCR) or fluorescent in situ hybridization (FISH), which proves especially helpful in diagnosing extra-abdominal tumors (that have been previously identified in pleural, intrathoracic, posterior cranial fossa, soft tissue, bones, ovarian, and sinonasal spaces) [6].

Published studies have varied widely with regard to the type and sequence of treatment regimens utilized (i.e., chemotherapy, surgery, radiation, and stem cell transplant (SCT)). Overall survival (OS) rates range from 26 to 64% at 3 years and 10 to 33% at 5 years [7–13]. Median OS range from 13 to 60 months [7–13]. DSRCT has demonstrated to be both alkylator-sensitive and dose-responsive to chemotherapeutic agents. Most institutions tend to use a treatment protocol similar to that of Ewing sarcoma due to the similarities observed in genetics, age, gender, and primitive appearance of neoplastic cells. The addition of whole abdominopelvic radiation (WAP) to multimodal therapy has resulted in median survival of 37.7 and 48 months; one study found postoperative WAP to be predictive of improvement in 3-year OS [14, 15]. Published studies differ in definitions of aggressive surgery, for example, removal of >90% of the tumor, all visible tumors, or leaving no more than 2 cm of tumor. Regardless, it has been shown that more complete surgery results in statistically significant prolonged OS, 49 months versus 12 months (*p*=0.004) [9]. Recent studies indicate promising survival statistics when cytoreductive surgery is used in combination with hyperthermic intraperitoneal chemotherapy (HiPEC), an emerging treatment modality in various peritoneal sarcomatosis such as DSRCT, rhabdomyosarcoma, leiomyosarcoma, gastrointestinal stromal tumors (GISTs), and liposarcoma [3, 16]. A phase 2 trial of complete resection, HiPEC, and WAP had 3-year overall survival of 79% [17].

Perhaps as a consequence of the dearth of research involving DSRCT, there has not been a validated method of stratifying patients into different risks and/or prognostication categories. One example of prognostic indicator is that, the presence of extra-abdominal metastases may portend a poor prognosis with median OS of 40 months without metastasis and 16 months with metastasis (*p*=0.21) [16]. Bertuzzi et al. identified several factors associated with disease recurrence including “metastatic disease to sites other than lung, tumor volume >100 mL, axial site involvement, lack of response to first-line therapy, and relapsed disease” [18]. Such heterogeneity in the available literature necessitates a further exploration of the outcomes of recently published high-intensity treatment regimens consisting of chemotherapy, radiation, surgical excision, and SCT.

Since 1996, the Children's Hospital at Montefiore (CHAM) has utilized a standard DSRCT treatment regimen that begins with a four-pronged approach of surgery, P6 chemotherapy (an intense alkylator-based induction regimen), and SCT, which is later followed by WAP. We performed a comprehensive review of the literature to help further elucidate the current landscape of DSRCT treatment.

## 2. Materials and Methods

### 2.1. Objectives

The primary objective of this study was to compare the outcome of SCT patients with that of non-SCT patients in the literature. We hypothesized that SCT would prolong OS when compared to regimens without SCT. Secondary objectives included adding cases of patients with DSRCT treated at CHAM from 1996 to 2013 to the literature and confirming published prognostic indicators.

### 2.2. Case Studies

Study population included any patient who received treatment at CHAM with histologic diagnosis of DSRCT. Data were collected on patients' treatment regimens, disease-free survival (DFS) rate, and OS rate. All pediatric and adult patients with DSRCT who present to any Montefiore-associated hospital are treated by the Pediatric Oncology Division at CHAM. The electronic medical record at CHAM was searched using the Clinical Looking Glass tool for all patients with a diagnosis of DSRCT. The study was approved by the Institutional Review Board at Montefiore.

Inclusion criteria consisted of those patients with histologically confirmed diagnosis of DSRCT who received ≥1 treatment at CHAM between the years 1996 and 2013. These treatments included debulking surgery (removal of >90% of tumor), cytoreductive surgery (resection of tumor to a visible size of <1.0 cm), SCT, abdominal radiation, chemotherapy (neoadjuvant or adjuvant), and immunotherapy.

Those patients who only presented to CHAM for consultation but were not treated within our institution were excluded. Additionally, those patients who had <6 months of follow-up time were not included in the study.

### 2.3. Literature Review

A search of the electronic databases PubMed, Cochrane Central Register of Controlled Trials (CENTRAL), MEDLINE, and EMBASE for the terms “desmoplastic” AND “small” AND “round” AND “cell” AND “tumor” was performed. Additionally, the bibliographies of the chosen articles were hand-searched in an effort to capture any additional published and unpublished trials.

Published reports of 1-2 cases were felt to represent a positive publication bias; thus, a study was only included if it included ≥3 cases of histologically confirmed DSRCT. Studies that contained general descriptions of patient populations but did not individually identify patients and their respective disease course (namely, last known event and time to last known event) were excluded. Any publications from the same institution were compared to ensure that each patient was represented only once; if a patient's demographics were similar to those of another patient, then the older data were excluded for that patient.

### 2.4. Statistical Analysis

Data were collected from the electronic medical records at CHAM. Potential confounders to the success of SCT included the presence of extra-abdominal metastasis and the extent of surgery (cytoreductive versus debulking), which have previously been published in the literature as having statistically significant prognostic implications. Patient demographics were tabulated using mean, median, and mode for continuous variables and percentages for categorical variables. The statistical methods used to evaluate the study objectives included Kaplan–Meier to estimate survival and log-rank to compare OS and DFS. OS was calculated as date of diagnosis to date of death or last follow-up.

## 3. Results and Discussion

### 3.1. Case Studies

For the four CHAM patients, the average follow-up was 58 months. One patient died of disease at 39 months after diagnosis, one patient remains alive with relapsed disease at 40 months, and two patients have no evidence of disease at 60 and 91 months. An overview of these patients' treatment courses can be seen in Table 1.

Patient #1 initially presented with decreased appetite, worsening asthma, weight gain, and left-sided pain. Computed tomography (CT) scan was concerning for an ovarian tumor. An oophorectomy revealed DSRCT. The patient underwent P6 chemotherapy (consisting of alternating cycles of cyclophosphamide, doxorubicin, and vincristine with ifosfamide and etoposide), debulking surgery, consolidation therapy with high-dose chemotherapy, autologous SCT, and WAP. The patient is alive and disease free at 91 months.

Patient #2 presented with bright red blood per rectum with a normal colonoscopy, followed by abdominal pain and vomiting. CT scan revealed abdominal masses in the right lower quadrant and another mass in the perirectal area. CT-guided biopsy provided a diagnosis of DSRCT. The patient's treatment course consisted of P6 chemotherapy, debulking surgery, SCT, and partial course of radiation. The patient's course was complicated by colonic perforation with hemicolectomy and ostomy placement, followed by bowel reanastamosis with wound infection. The patient relapsed at 32 months after diagnosis and is currently still alive receiving treatment at 48 months after diagnosis.

Patient #3's course began with P6 chemotherapy. Tumor resection included distal pancreatectomy, splenectomy, and transverse colon resection with colonic anastamosis. The patient then underwent SCT followed by radiation. The course was complicated by surgical evacuation of an abdominal hematoma. The patient was alive and disease free at 60 months after diagnosis.

Patient #4 had unilateral ear pain followed by development of stiff neck. Magnetic resonance imaging (MRI) was done showing inflammation of the spine; at the same time, a chest X-ray (CXR) showed a lesion in the spine. CT abdomen/pelvis showed a right kidney mass, and a bone scan was positive in the sternum, spine, and head. Bone marrow biopsy showed DSRCT. Treatment began with P6 chemotherapy, followed by tumor resection with radical nephrectomy and retroperitoneal lymph node dissection. The patient then underwent autologous SCT and radiation. The patient relapsed 20 months after diagnosis. The patient eventually passed away from metastatic disease approximately 30 months after completing treatment.

### 3.2. Literature Review

#### 3.2.1. Demographics

The systematic review of the literature revealed 512 publications in the literature (Supplementary Figure S1). A total of 492 publications were excluded for lack of OS data (*n*=7), non-DSRCT diagnosis (*n*=231), ≤2 patients (*n*=249), or a single-agent study (*n*=5), which left a remainder of 20 published papers; these included 279 patients in the literature including the patients treated at CHAM (Supplemental Table S1). Within that population, 23 were omitted for lack of OS data (*n*=17), initial misdiagnosis (*n*=2), palliative treatment only (*n*=2), and suspected overlap with other patients (*n*=2). A total of 256 patients were available for our statistical analysis (Table 2).

The median age was 18.3 years (range 2–64 years) with 86% male patients and 13% female patients. With regard to the primary site of disease, 77% (198) had primary abdominal/pelvic site and 9% (24) had nonabdominal/pelvic site, while 13% (34) were unknown. Liver metastasis was present in 126 (49%) patients. There was reported extra-abdominal metastasis in 62 patients (24%), while 55% did not have extra-abdominal metastasis. The remainder of the patients did not have this information available in the publication.

Of the 256 patients, 71 patients (28%) had SCT as part of their treatment regimen. Of the 71 patients who underwent SCT, 23 (32%) were not in remission, 13 (18%) were in remission, and 35 (49%) had unknown status at time of remission. There were 86 (34%) patients who had radiation and 167 (65%) patients who did not undergo radiation. Sixteen patients (6%) underwent HiPEC, while 202 (78%) did not. The remaining patients did not have this information clearly stated in the publication. Within the various treatment regimens, 130 (51%) patients received doxorubicin and 15 (6%) had regimens that did not contain doxorubicin. The other patients' chemotherapy regimens were not clearly defined whether they contained doxorubicin. As part of their chemotherapy regimen, 55% of patients received an alkylating agent, and 1% reported no alkylating agent; the remainder of the patients did not have this number reported. The number of total treatments (e.g., chemotherapy, radiation, SCT, surgery, or HiPEC) per patient was reported as 0 (3.5%), 1 (20%), 2 (34%), 3 (25%), and 4-5 (14%). For surgical excision, 22% were reported as complete and 15% were reported as incomplete. Surgical information was not available for the remainder of the patients.

#### 3.2.2. Outcomes

For all patients, the 3-year OS was 36% and 5-year OS was 13% (Table 3). There was not enough information in the literature to calculate DFS for patients, as most studies reported OS only.

#### 3.2.3. Treatment

**Figure 1:**
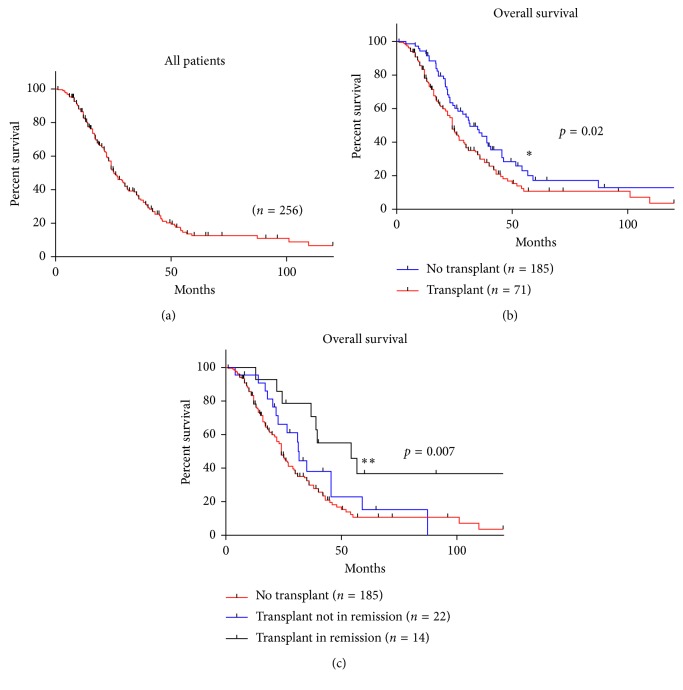
(a) The Kaplan–Meier curve of all patients included in the data analysis. (b) The Kaplan–Meier curve of patients who did not receive a stem cell transplant and patients who received a stem cell transplant. (c) The Kaplan–Meier curve of patients who did not receive a stem cell transplant, patients who received a stem cell transplant not in remission, and patients who received a stem cell transplant in remission.

A Kaplany–Meier curve of the OS of 256 patients whose information was available for our statistical analysis is shown in Figure 1(a).  Figure 1(a). Those with SCT (*n*=71) had an improved OS compared to those with no SCT (*n*=185,  *p*=0.02; Figure 1(b)). There was a statistically significant difference in OS between no transplant (*n*=185), SCT in remission (*n*=13), and SCT not in remission (*n*=13, *p*=0.007) (Figure 1(c)), as well as between no transplant (*n*=185) and SCT in remission (*n*=14, *p*=0.004) seen in Supplementary Figure 2A. No difference was seen in comparing no transplant (*n*=185) with transplant not in remission (*n*=23, *p*=0.07) (Supplementary Figure 2B). Finally, there was no difference between SCT not in remission (*n*=22) and transplant in remission (*n*=14, *p*=0.07) (Supplementary Figure 2C).

**Figure 2:**
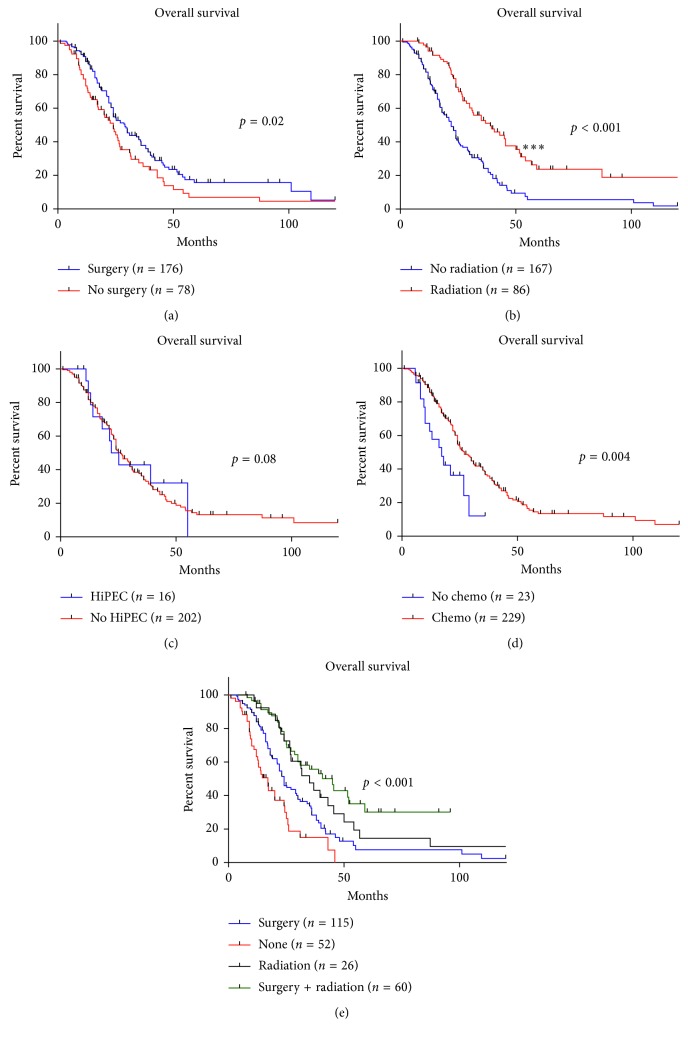
(a) The Kaplan–Meier curve of patients who had surgery and patients who did not have surgery. (b) The Kaplan–Meier curve of patients who did not receive radiation and patients who received radiation. (c) The Kaplan–Meier curve of patients who received hyperthermic intraperitoneal chemotherapy and patients who did not receive hyperthermic intraperitoneal chemotherapy. (d) The Kaplan–Meier curve of patients who received chemotherapy and patients who did not receive chemotherapy. (e) The Kaplan–Meier curve of patients who received surgery, patients who received radiation, patients who received radiation and surgery, and patients who did not receive radiation or surgery. HiPEC = hyperthermic intraperitoneal chemotherapy; chemo = chemotherapy.

Patients who underwent more thorough surgical excision had improved OS compared to those who underwent incomplete or no surgery (*p*=0.02) (Figure 2(a)). Radiation also improved OS (*p* < 0.0001) (Figure 2(b)). HiPEC therapy did not provide a statistically significant improvement in OS (*p*=0.08) (Figure 2(c)). Chemotherapy did demonstrate an improved OS (*p*=0.004) (Figure 2(d)), though using a regimen with doxorubicin did not (*p*=0.12) (Supplementary Figure 3A). Finally, those who underwent surgery and radiation had an improved OS compared to either treatment alone or neither treatment (*p* < 0.001) (Figure 2(e)).

#### 3.2.4. Prognostic Indicators

**Figure 3:**
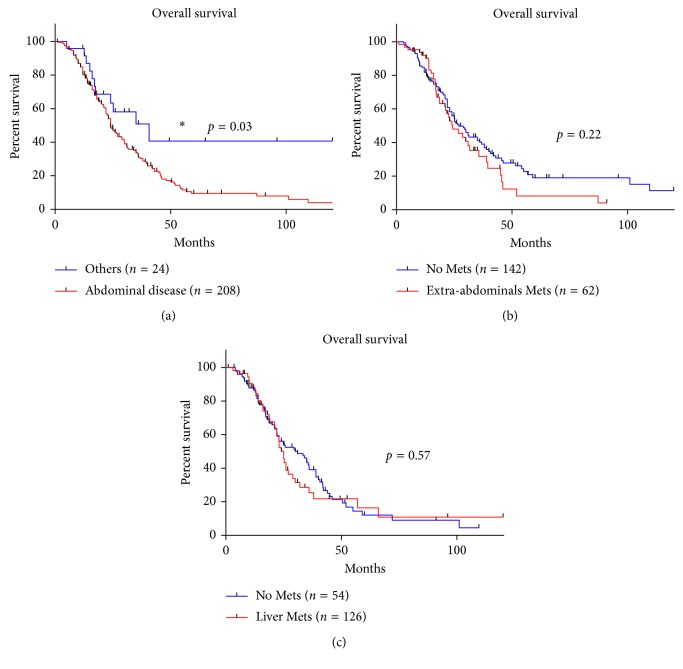
(a) The Kaplan–Meier curve of patients whose primary site of disease was in the abdomen and patients whose primary site of disease was outside of the abdomen. (b) The Kaplan–Meier curve of patients who had extra-abdominal metastasis at time of diagnosis and patients who did not have extra-abdominal metastasis at time of diagnosis. (c) The Kaplan–Meier curve of patients who had liver metastasis at time of diagnosis and patients who did not have liver metastasis at time of diagnosis. Mets = metastasis.

Several prognostic indicators were compared to those previously reported in the literature. We found that the presence of primary disease outside of the abdomen had an improved OS (*p* < 0.03), as seen in Figure 3(a). We also found that those who had extra-abdominal metastasis or liver metastasis did not have a statistically significant worsening of their OS versus patients without those characteristics (*p*=0.22 and *p*=0.57, resp.; Figures 3(b) and 3(c)).

## 4. Conclusions

Our review of over 250 patients in the literature with DSRCT demonstrates that treatment with SCT in remission, complete surgical resection, and radiation had statistically significantly improved OS at 3 and 5 years, compared to the patients who were not in remission at the time of SCT, incomplete or no surgical resection, and no radiation, respectively. The results of this study postulate that more aggressive multimodal treatment (consisting of extensive surgical resection, chemotherapy, and/or radiation) that achieves remission, followed by SCT, could result in improved long-term outcomes for these patients. A prospective study would be required to accurately assess whether SCT in this patient population is necessary; patients with DSRCT in remission may fare as well without a SCT.

The last major published review of the DSRCT literature by Kretschmar et al. in 1996 included 101 patients, for which median survival was 17 months (range 3–72 months) with only two patients disease free beyond 2 years at 40 and 48 months [1]. Our large-scale review contributes to the literature in that it represents more current treatment regimens and improved OS compared to the prior time period.

Multimodal aggressive treatment portends a survival advantage in our study and may benefit from further prospective evaluation. A previous publication showed that chemotherapy, surgery, and radiation therapy together produced a 55% OS at 3 years versus 27% if one of these therapeutic modalities was missing [13]. In another study, there was a median survival of 34 months in patients who had complete surgical resection with systemic chemotherapy versus 14 months for chemotherapy alone [19].

Since those who were in remission at the time of SCT fared the best, SCT may be a possible consideration for those who respond to first-line chemotherapy. A previous study by Fraser et al. published data on SCT in a variety of patients, including four patients with DSRCT [20]. When these patients were in complete remission, the 3-year OS was 76% as compared to 27% for those in partial remission (*p*=0.08). Thus, SCT may be most beneficial in a select group of DSRCT that has no evidence of disease.

We investigated prognostic indicators that were previously published in the literature. In our study, we demonstrate that the presence of primary disease outside of the abdomen had an improved OS compared to intra-abdominal disease, which has not been previously reported. This may be due to the containment of the tumor allowing for complete surgical resection, as in testicular disease. Those who had extra-abdominal metastasis or liver metastasis showed no worsening in OS, which was different from previously published studies [6, 14]. This distinction may be attributable to differences in treatment with more patients receiving multimodal therapy.

In addition, the efficacies of other treatment options (surgery, chemotherapy, and radiation) were investigated. In one study, incomplete cytoreduction was associated with no patient surviving >15 months [21]. Lal et al. demonstrated the necessity for completeness of surgical excision. While patients who underwent gross tumor resection had an OS of 58% at 3 years, there was 0% OS in patients where visible deposits remained [13]. Our study also indicated that more complete surgical excision results in improved overall outcome, thus substantiating that having tumor burden amenable to complete resection improved survival.

Due to the heterogeneity of treatment in the literature, we did not find statistically significant advantages in using a doxorubicin-containing chemotherapy regimen or HiPEC. Like HiPEC, radiotherapy is generally indicated for local disease confined to the abdomen; thus, it would not be feasible for patients with larger tumor burden [22]. Patients in our study who underwent radiation as part of multimodal therapy had improved OS. This finding may be biased by selection of only those patients with minimal tumor burden or functional status to be able to tolerate the treatment.

There are several limitations to our study. Studies that did not report individual patient outcomes or grouped DSRCT with other soft tissue sarcomas (STSs) were excluded. Our results are similar to those of studies consisting of aggregated DSRCT data in the literature. For example, a study conducted by Zhang et al. analyzed a cohort of 48 patients with DSRCT and found median overall survival of 24 months; the variables associated with improved OS included “surgery, effective debulking surgery, chemotherapy, and any two or more combined therapeutics” (*p* < 0.05). There was also a Cochrane Review that examined SCT in nonrhabdomyosarcoma STS, within which 27 patients carried a diagnosis of DSRCT [9, 23]. The graph of OS of the 27 patients with DSRCT appeared similar to the graph of the 80 total patients, but there was no direct statistical comparison. A randomized control trial (RCT), which included multiple STS histologies, did not show a significant difference in OS between the SCT and non-SCT groups; the authors concluded that there was some evidence that there might not be improved survival after SCT [24]. Another RCT by Bui-Nguyen et al. had 87 STS patients, 2 of which had DSRCT. In their population, patients who had achieved a complete response prior to SCT had a longer OS compared to all patients. However, the findings of these studies are difficult to extrapolate as they involve multiple histologies with variable chemosensitivity.

Other limitations of this study are consistent with the methodologies of a literature review and the potential for reporting bias. We were unable to complete a multivariate analysis, systematic review, or meta-analysis due to our limited number of patients and lack of published details on individual patients; conclusions that could be reached from these analyses are beyond the scope of this article. Future analyses would benefit from a centralized registry of DSRCT patients to aggregate individual patient data. Treatment regimens varied widely among the included patient population. A total of 26 different chemotherapy regimens are represented in this review. Furthermore, the order of all treatment regimens (chemotherapy, radiation, surgery, and SCT) amongst all studies was not uniform. Lastly, the review of the literature cannot always discern the tolerability of the treatment. As such, variability in the treatment may reflect toxicity rather than differences in the planned treatment.

This review of the literature supports additional investigation of SCT as part of the multimodal treatment for patients who are able to achieve remission. Further research is warranted to validate these findings in this narrow population. Complete resection (and by extension, likely more limited disease) has consistently been reported to be most important facet of multimodal treatment. Likewise, the role of HiPEC in a select group of DSRCT may need to be further explored. Given the poor prognosis, there are several ongoing clinical trials investigating other agents, such as Irinotecan, Temozolomide and Bevacizumab in Combination with Existing High Dose Alkylator Based Chemotherapy and Phase I Trial of Intraperitoneal Radioimmunotherapy with ^131^I-8H9 [25, 26]. Shukla et al. found no consistent targetable mutations found in DSRCT samples using Sequenom analysis, but perhaps whole genome sequencing may lead to identification of other potential targets of transcriptional dysregulation [27]. Further research targeting the *EWS/WT1* translocation (as currently being explored in early phase trials of the Ewing sarcoma) may provide promise in this difficult to treat disease.

## Figures and Tables

**Table 1:** Patients with desmoplastic small round cell tumor treated at Children's Hospital at Montefiore.

Patient	Age at diagnosis	Gender	Date of diagnosis	Date of relapse	Date of last follow-up	Status at last follow-up	Months to last follow-up	1st treatment	2nd treatment	3rd treatment	4th treatment	5th treatment
1	16	Female	5/8/2008		12/8/2015	Alive, disease free	91	Oophorectomy	P6 chemotherapy	Surgery at MSKCC	SCT	Total abdominal radiation
2	21	Male	9/24/2012	6/1/2015	9/24/2016	Alive, relapsed	40	Resection of abdominal tumor	P6 chemotherapy	Surgery at MSKCC	SCT	Partial course of radiation
3	11	Male	11/16/2010		11/16/2015	Alive, disease free	60	Vincristine, dactinomycin, cyclophosphamide	Tumor resection	SCT	Radiation	
4	19	Male	1/9/2008	9/28/2009	4/27/2011	Death from metastatic disease	39	P6 chemotherapy	Tumor resection	SCT	Radiation	

MSKCC: Memorial Sloan Kettering Cancer Center; SCT: stem cell transplant.

**Table 2:** Demographics of patients with desmoplastic small round cell tumor treated at Children's Hospital at Montefiore and in literature review.

All patients		
Median age at diagnosis	18.3 years	
Overall survival	3 years, 36%	5 years, 13%

*Category*	*N*	*Percentage*
Female	34	13
Male	220	86
Unknown	2	1

Primary abdominal/pelvic disease	198	77
Primary nonabdominal disease	24	9
Unknown	34	13

Liver metastasis	126	49
No liver metastasis	54	21
Unknown	76	30

Extra-abdominal metastasis	62	24
No extra-abdominal metastasis	142	55
Unknown	52	20

SCT	71	28
SCT not in remission	23	
SCT in remission	13	
SCT unknown remission	35	
No SCT	185	72

Radiation	86	34
No radiation	167	65
Unknown	3	1

HiPEC	16	6
No HiPEC	202	79
Unknown	38	15

Doxorubicin	130	51
No doxorubicin	15	6
Unknown	111	43

Alkylating agent	134	52
Alkylating-like agent	8	3
No alkyating agent	3	1
Unknown	111	43

0 treatment	9	4
1 treatment	52	20
2 treatments	87	34
3 treatments	65	25
4–5 treatments	38	15
Unknown	4	2

Complete surgical excision (2,3,4)	56	22
Incomplete surgical excision (0,1)	39	15
Unknown	161	63

SCT: stem cell transplant; HiPEC: hyperthermic intraperitoneal chemotherapy.

**Table 3:** Overall survival for all patients, patients with stem cell transplant, patients with stem cell transplant not in remission, patients with stem cell transplant in remission, and patients with no stem cell transplant.

	3-year overall survival	5-year overall survival
All	36%	13%
SCT^ab^	45%	17%
SCT not in remission^b^	38%	15%
SCT in remission^bc^	71%	37%
No SCT^ac^	30%	11%

SCT: stem cell transplant. ^a^SCT versus no SCT (*p*=0.02); ^b^SCT versus SCT not in remission versus SCT in remission (*p*=0.007); ^c^SCT in remission versus no SCT (*p*=0.004). The remainder of *p* values were not significant.

## Abbreviations

DSRCT: Desmoplastic small round cell tumor
OS: Overall survival
SCT: Stem cell transplant
CHAM: Children's Hospital at Montefiore
WAP: Whole abdominopelvic radiation
*WT1*: Wilms' tumor gene
*EWS*: Ewing's sarcoma gene
HiPEC: Hyperthermic intraperitoneal chemotherapy
GIST: Gastrointestinal stromal tumor
MRI: Magnetic resonance imaging
CT: Computed tomography
CXR: Chest X-ray
STS: Soft tissue sarcoma
RCT: Randomized control trial
PCI: Peritoneal cancer index
Mets: Metastasis
PCR: Polymerase chain reaction
FISH: Fluorescent in situ hybridization.

## Supplementary Materials

Supplementary Materials Figure S1: flow chart of literature review. A total of 492 publications were excluded for lack of OS data (*n*=7), non-DSRCT diagnosis (*n*=231), ≤2 patients (*n*=249), or a single-agent study (*n*=5), which left a remainder of 20 published papers; these included 279 patients in the literature including the patients treated at CHAM. Within that population, 23 were omitted for lack of OS data (*n*=17), initial misdiagnosis (*n*=2), palliative treatment only (*n*=2), and suspected overlap with other patients (*n*=2). Table S1: all studies included in literature review. The 20 published papers in the literature comprised 275 patients. Figure S2A: the Kaplan–Meier curve of patients who had a stem cell transplant in remission and patients who did not have a stem cell transplant. Figure S2B: the Kaplan–Meier curve of patients who did not have a stem cell transplant and patients who had a stem cell transplant not in remission. Figure S2C: the Kaplan–Meier curve of patients who had a stem cell transplant in remission and patients who had a stem cell transplant not in remission. There was a statistically significant difference in OS between no transplant (*n*=185), SCT in remission (*n*=13), and SCT not in remission (*n*=13, *p*=0.007) (Figure 1(c)), as well as between no transplant (*n*=185) and SCT in remission (*n*=14, *p*=0.004) seen in Figure S2A. No difference was seen in comparing no transplant (*n*=185) with transplant not in remission (*n*=23, *p*=0.07) (Figure S2B). Finally, there was no difference between SCT not in remission (*n*=22) and transplant in remission (*n*=14, *p*=0.07) (Figure S2C). Figure S3: the Kaplan–Meier curve of patients who had doxorubicin as part of their chemotherapy regimen and patients who did not have doxorubicin as part of their regimen. Chemotherapy did demonstrate an improved OS (*p*=0.004), though using a regimen with doxorubicin did not (*p*=0.12).
