# Supplementary material for: High-Dose Chemotherapy with Stem Cell Rescue in Desmoplastic Small Round Cell Tumor: A Single-Institution Experience and Review of the Literature
Source: Sarcoma. 2018 May 6;2018:1948093. doi: 10.1155/2018/1948093 (PMC5960572; doi:10.1155/2018/1948093)
Supplement: Supplementary Materials — Figure S1: flow chart of literature review. A total of 492 publications were excluded for lack of OS data (n=7), non-DSRCT diagnosis (n=231), ≤2 patients (n=249), or a single-agent study (n=5), which left a remainder of 20 published papers; these included 279 patients in the literature including the patients treated at CHAM. Within that population, 23 were omitted for lack of OS data (n=17), initial misdiagnosis (n=2), palliative treatment only (n=2), and suspected overlap with other patients (n=2). Table S1: all studies included in literature review. The 20 published papers in the literature comprised 275 patients. Figure S2A: the Kaplan–Meier curve of patients who had a stem cell transplant in remission and patients who did not have a stem cell transplant. Figure S2B: the Kaplan–Meier curve of patients who did not have a stem cell transplant and patients who had a stem cell transplant not in remission. Figure S2C: the Kaplan–Meier curve of patients who had a stem cell transplant in remission and patients who had a stem cell transplant not in remission. There was a statistically significant difference in OS between no transplant (n=185), SCT in remission (n=13), and SCT not in remission (n=13, p=0.007) (Figure 1(c)), as well as between no transplant (n=185) and SCT in remission (n=14, p=0.004) seen in Figure S2A. No difference was seen in comparing no transplant (n=185) with transplant not in remission (n=23, p=0.07) (Figure S2B). Finally, there was no difference between SCT not in remission (n=22) and transplant in remission (n=14, p=0.07) (Figure S2C). Figure S3: the Kaplan–Meier curve of patients who had doxorubicin as part of their chemotherapy regimen and patients who did not have doxorubicin as part of their regimen. Chemotherapy did demonstrate an improved OS (p=0.004), though using a regimen with doxorubicin did not (p=0.12). [file 1948093.f1.pdf]

**512 Publications Reviewed**	
	**492 Publications Omitted for:**
	≤ 2 patients (n=249)
	Not DSRCT diagnosis (n=231)
	No individual OS data (n=7)
	Single agent study (n=5)
**20 Publications**	
n=279 patients	
	**n=23 Patients Omitted for:**
	No OS data (n=17)
	Initial misdiagnosis (n=2)
	Palliative treatment only (n=2)
	Suspected overlap (n=2)
**n=256 Patients**	
SCT[1] patients (n=79)	No SCT (n=185)

SUPPLEMENTARY FIGURE S1. Flow Chart of Literature Review

[1] SCT = Stem Cell Transplant

A

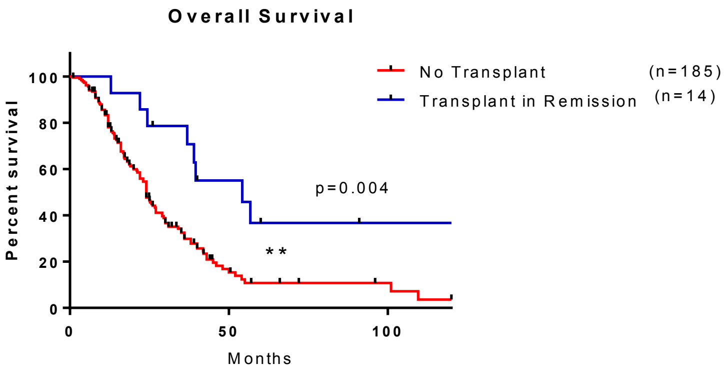

B

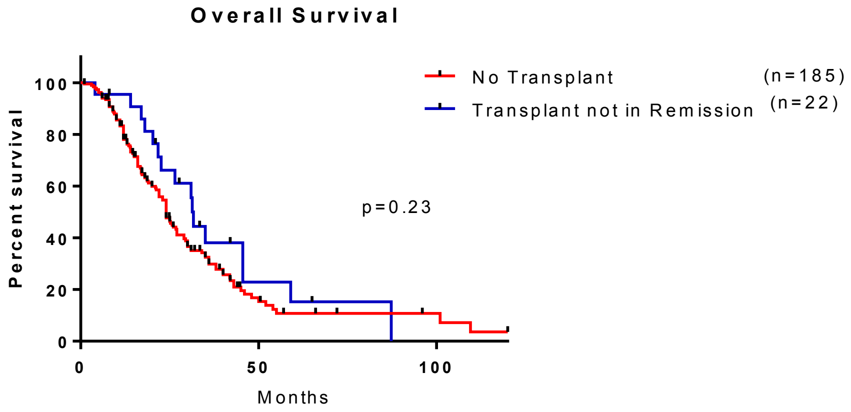

C

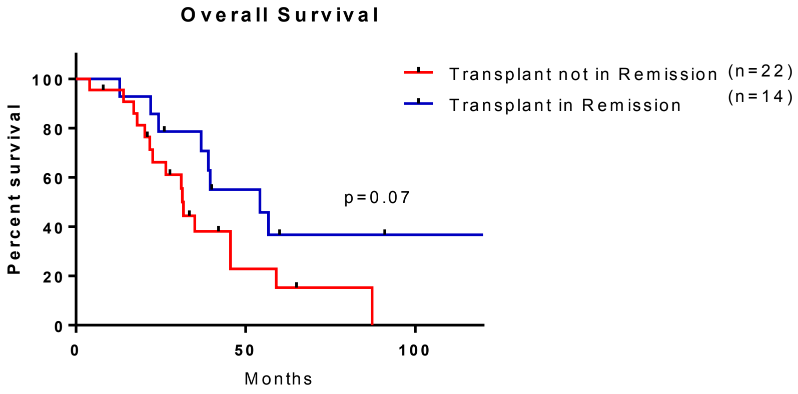

SUPPLEMENTARY FIGURE 2 A Kaplan-Meier curve of patients who had Stem Cell Transplant in remission and patients who did not have Stem Cell Transplant. B . Kaplan-Meier curve of patients who did not have Stem Cell Transplant and patients who had a Stem Cell Transplant not in remission. C Kaplan-Meier curve of patients who had Stem Cell Transplant in remission and patients who had a Stem Cell Transplant not in remission.

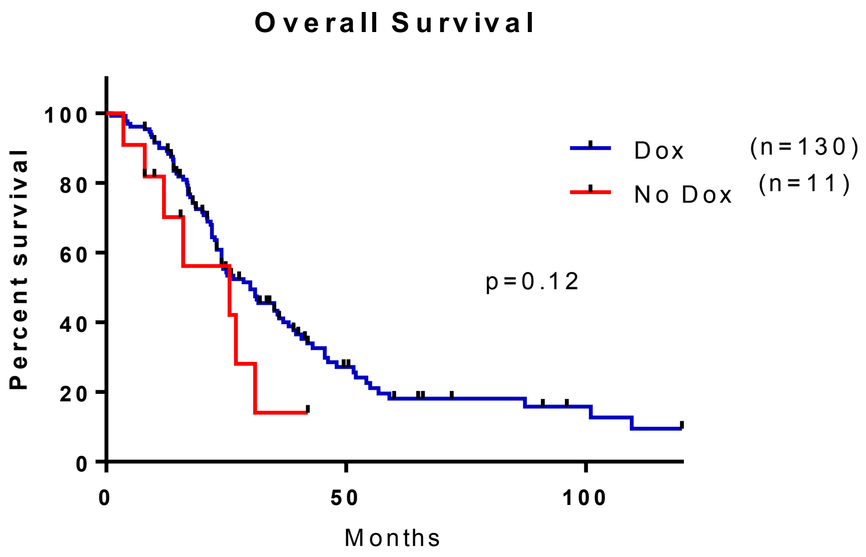

SUPPLEMENTARY FIGURE S3 Kaplan-Meier curve of patients who had doxorubicin as part of their chemotherapy regimen and patients who did not have doxorubicin as part of their regimen. (Dox = doxorubicin)

Author	Year	Includes some SCT[1] patients	Number of Patients In Paper
Gerald	1991	no	19
Parkash	1995	no	3
Farhat	1996	no	5
Ordonez	1998	no	39
Kurre	2000	no	3
Liang	2000	no	3
Gil	2004	no	7
Msika	2010	no	3
Tang	2015	no	18
Zhang	2015	no	11
Kretschmar	1996	yes	3
Kushner	1996	yes	12
Cummings	1997	yes	6
Lae	2002	yes	32
Livaditi	2006	yes	5
Saab	2007	yes	11
Bisogno	2010	yes	14
Hayes-Jordan	2010	yes	24
Philippe	2012	yes	38
Forlenza	2015	yes	19
CHAM	2016	yes	4
**Total**			**279**

SUPPLEMENTARY TABLE S1 All Studies Included in Literature Review
[1]SCT is Stem Cell Transplant.
